# Torsion and Antero-Posterior Bending in the *In Vivo* Human Tibia Loading Regimes during Walking and Running

**DOI:** 10.1371/journal.pone.0094525

**Published:** 2014-04-14

**Authors:** Peng-Fei Yang, Maximilian Sanno, Bergita Ganse, Timmo Koy, Gert-Peter Brüggemann, Lars Peter Müller, Jörn Rittweger

**Affiliations:** 1 Key Laboratory for Space Bioscience and Biotechnology, School of Life Sciences, Northwestern Polytechnical University, Xi'an, China; 2 Division of Space Physiology, Institute of Aerospace Medicine, German Aerospace Center, Cologne, Germany; 3 Institute of Biomechanics and Orthopaedics, German Sport University Cologne, Cologne, Germany; 4 Department of Orthopaedic and Trauma Surgery, University of Cologne, Cologne, Germany; 5 Institute for Biomedical Research into Human Movement and Health, Manchester Metropolitan University, Manchester, United Kingdom; University of Utah, United States of America

## Abstract

Bending, in addition to compression, is recognized to be a common loading pattern in long bones in animals. However, due to the technical difficulty of measuring bone deformation in humans, our current understanding of bone loading patterns in humans is very limited. In the present study, we hypothesized that bending and torsion are important loading regimes in the human tibia. *In vivo* tibia segment deformation in humans was assessed during walking and running utilizing a novel optical approach. Results suggest that the proximal tibia primarily bends to the posterior (bending angle: 0.15°–1.30°) and medial aspect (bending angle: 0.38°–0.90°) and that it twists externally (torsion angle: 0.67°–1.66°) in relation to the distal tibia during the stance phase of overground walking at a speed between 2.5 and 6.1 km/h. Peak posterior bending and peak torsion occurred during the first and second half of stance phase, respectively. The peak-to-peak antero-posterior (AP) bending angles increased linearly with vertical ground reaction force and speed. Similarly, peak-to-peak torsion angles increased with the vertical free moment in four of the five test subjects and with the speed in three of the test subjects. There was no correlation between peak-to-peak medio-lateral (ML) bending angles and ground reaction force or speed. On the treadmill, peak-to-peak AP bending angles increased with walking and running speed, but peak-to-peak torsion angles and peak-to-peak ML bending angles remained constant during walking. Peak-to-peak AP bending angle during treadmill running was speed-dependent and larger than that observed during walking. In contrast, peak-to-peak tibia torsion angle was smaller during treadmill running than during walking. To conclude, bending and torsion of substantial magnitude were observed in the human tibia during walking and running. A systematic distribution of peak amplitude was found during the first and second parts of the stance phase.

## Introduction

Evidence suggests that both the geometry and thus the mechanical properties of long bones adapt to the mechanical load they are exposed to [1]–[3]. In the absence of an easy way to assess *in vivo* mechanical loads acting on bones, bone geometry, which is deemed to be causally related to its loading history, has been taken to predict the *in vivo* bone loading history [4], [5]. For instance, by analyzing a stack of peripheral quantitative computed tomography (pQCT) images taken across the human tibia, it was concluded that the almost circular distal tibia seems to be adapted to compressive loading patterns, while the non-circular geometry of the proximal tibia is the result of increased torsion and bending [5]. However, there are several problems with this approach [6]. These include a lack of absolute values of cross-sectional geometric properties and a potential misalignment between the loading history and bone cross-sectional geometry [6]. It is obvious, and not only therefore, that accurate measurements of real-world *in vivo* bone loading patterns are needed.

Obtaining the information of the bone loading patterns is very important to better understand bone's mechano-adaptation, as bone responds differently to different deformation patterns, *e.g.* to torsion or compression [7]. Evidence also suggests that bone formation varies between anatomical sites due to the uneven local strain distribution and deformation patterns, as illustrated across the loaded ulna in rats [8]. Likewise, bending load, rather than local pressure, was capable of creating substantial periosteal mineral apposition in rats [9]. Moreover, understanding *in vivo* bone deformation is clinically relevant, in particular in relation to fatigue fracture. For example, *ex vivo* evidence suggests that mixed-mode loading is associated with greater bone fragility than uniaxial loading [10]. Similarly, changing the loading mode from pure compression to a combination of torsional and compressive loading facilitates propagation of microcracks within the bone [11], and bones are relatively stronger when loaded by habitual load patterns than when exposed to novel loading regimes [12].

The *in vivo* bone deformation data currently available in literature mainly originates from studies that have used surgically implanted strain gauges. As noted previously, at least three strain gauges have to be attached around the long bone shaft to determine the neutral axis of bending and compute bending load or deformation. For most species, including humans, this operation is not feasible without affecting their regular muscle functions [6], [13].

**Figure 1:**
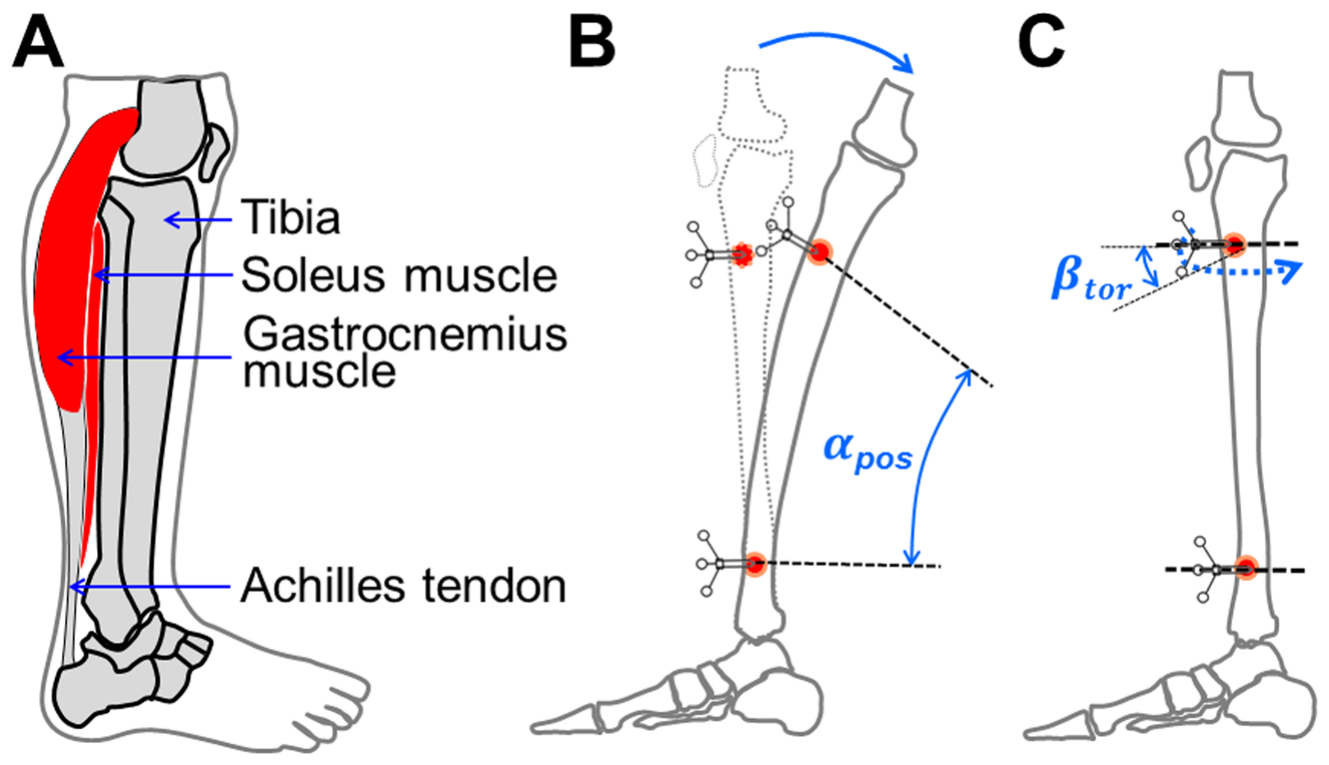
The demonstration of the human shank, the tibia posterior bending angle and torsion angle. A: anatomy of human shank. B: the demonstration of the posterior bending of the proximal tibia. *α_pos_* indicates the posterior bending angle. C: tibia torsion deformation. *β*_tor_ indicates the internal torsion angle whist the tibia is twisted.

Harold Frost's mechanostat theory explains the functional adaptation capability of bone to mechanical stimulation [14]. However, it is still under debate which loading parameters in terms of deformation type, amplitude, repetition cycles and frequency, are most effective for bone adaptation [15]. Furthermore, the major sources of force, as well as the deformation modes required to effectively maintain or regulate bone structure and metabolism, remain unclear. Bending moments have been approximated in mammals, *e.g.* horse, dog and goat, using paired strains gauges from opposite surfaces of bones, in cases where limb motion is mainly in the parasagittal plane [7], [16]–[18]. A series of classic studies on the bones of the lower extremities of animals in the 1980 s suggested that bending is occurring during different locomotor activities. This was demonstrated when strain gauge measurements showed that the anterior aspect of bone is under tension while the posterior aspect is under compression [16], [17], [19]–[21]. Recent studies in animal [18] and human models [22]–[24] suggest that bending is the primary component of long bone loading. The fact that most long bones are slightly curvatured also supports the idea that bending is notable, and that it may be enhanced by muscle contractions and the off-axis orientation of the bone to the center of body mass [25]. Furthermore, it has been speculated that the bone curvature is designed to improve the predictability of the bone load during different locomotor activities, since a curved bone is more likely to be bent than a straight bone [25]. Furthermore, studies have shown a shift of the bending neutral axis of long bones from the certroidal axis of the cross sectional area, indicating that long bones do indeed bend while experiencing axial loading [18], [26]. In addition, it was shown that the bone loading pattern changes throughout the stance phase of the gait cycle and varies with speed [27]–[29]. The underlying cause will be that muscles attached to bone change their moment with joint movement. For example, most muscle groups in the human shank insert into the posterior aspect of tibia or fibula (Figure 1A). Although these muscles work against poor lever arms, they still generate very large flexion moments [30], [31].

It is unclear in how far bending moments might be minimized by muscular contractions. Such contractions could protect the bone material and especially the long bones from bending stress accumulation, and therefore reduce fracture risks [32]–[35]. Muscle forces are thought by some to convert potential bending stress generated by reaction forces to compressive loading, which is less harmful for bone to tolerate [36]. An *in vivo* tibia strain study in humans seems to support the opinion. Milgrom *et al.* compared strain data in humans in fatigued and non-fatigued status [37]. The tensional strain of the antero-medial aspect of the tibia clearly increased when the gastrocnemius muscles were fatigue indicating that regular muscle activities might be crucial to maintain regular bone strains [37].

However, as noted above, one can apply paired or more gauges only where opposing sides of the bone are free of muscle insertions. Such an anatomical site is not available in any of large human long bones. To our knowledge, the *in vivo* bone loading patterns in humans during daily activities remains unknown.

In comparison to bending, the role of torsion on bone mechanical adaptation has received little to no attention. Several classic studies have demonstrated that the strain gradient is highly correlated with periosteal bone formation on different sites [26], [38]. If this holds true, then torsional loading would not be a crucial factor for periosteal bone formation, as torsional loading is only capable of generating relative small strain gradients for a near cylinder shaped long bone, compared to bending load. Contradictorily, some studies suggested that different constituents of the loading environment, namely axial loading and torsional loading, play a distinct role in regulating bone formation and structure [7], [39]. These studies indicated that torsional loading might be one of the essential components of the entire bone loading regimes. Likewise, it was found that torsion dominates mechanical loading of the femur and tibiotarsus of the emu during running and gait [40]. Of note, torsion seems to be the main determinant of the design of long bones in birds [26], [40]–[43]. To date, there is no salient evidence to suggest a strong role for torsion in the design of human long bones. One would intuitively assume that torsion is the driving loading pattern to maintain the almost circular cross-sectional geometry of long hollow bones [44], as torsion is capable of producing similar bone matrix deformation all along the circumference in different sites of the long bone. Results from an *in vivo* knee joint loading study indicated that the tibia-femur contact torsion moment was relatively small, with the normalized peak value (normalized by body weight times length) ranging from 0.53 to 1.1 [45]. However, considering the low capability of bone to resist torsional loading, we hypothesize that the human tibia may experience considerable torsional loading during walking and running.

Therefore, the goal of the present study was to use a novel optical segment tracking (OST) approach to investigate, for the first time, the *in vivo* human tibia loading regimes in terms of the tibia segment deformation regimes, including tibia antero-posterior (AP), medio-lateral bending angles (ML), torsion angles during most common locomotor activities for humans on the ground, *e.g.* walking and running. Furthermore, the relationship between the speed of walking and running, ground reaction force, moment and tibia deformation was assessed.

## Material and Methods

Five healthy male subjects (26–50 years old) were recruited to participate in this study. They were free of any muscle or joint injuries and had not undergone orthopedic surgery in the lower extremities within twelve months prior to the study.

### 1.1 Ethics statement

Written and oral explanation of the purposes, benefits and risks of the study procedure were given to the subjects at least 3 days before they signed the consent forms. The subjects have given written informed consent, as outlined in the PLOS consent form, to the publication of their photograph. This study has been approved by the two relevant Ethics Committees, namely the ethical committee of the North-Rhine Medical Board in Düsseldorf and the ethical committee of the Faculty of Medicine in the University of Cologne. The operations and experiments were performed at the Department of Orthopedic and Trauma Surgery of the University Hospital of Cologne.

### 1.2 OST approach for tibia segment deformation measurements

**Figure 2:**
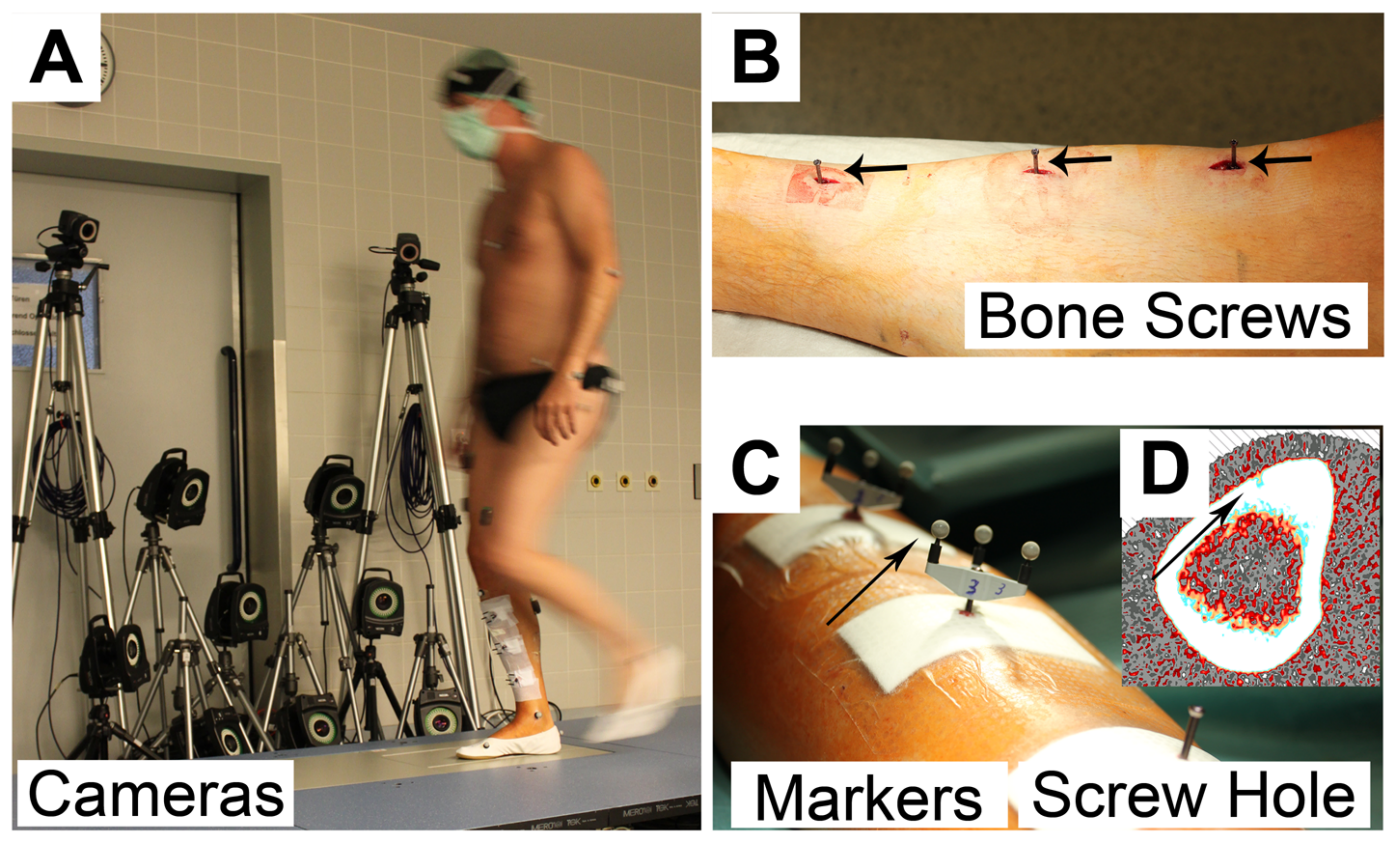
Illustration of the surgical details and the application of the OST approach in this study. A) The optical motion capture system with 8 high resolution cameras to track the retro-reflective markers affixed into tibial cortex, as well as two of the ten cameras for full body motion capturing; B) Implanted bone screws in the tibial cortex; C) Marker clusters were affixed to the endings of the bone screws; D) Cross-sectional pQCT image. The black arrow indicates the hole left behind after removal of the bone screw. OST: optical segment tracking.

A novel OST approach recently developed in our lab [46] has been adopted for tibia segment deformation recording in this study. Briefly, three mono-cortical bone screws were partially implanted into the anterior-medial aspect of the tibial cortex (Figure 2A–B). A marker cluster with a set of three non-collinear retro-reflective markers (Ø5 mm, Géodésie Maintenance Services, Nort Sur Erdre, France, Figure 2C) was mounted on each bone screw. The trajectories of the marker clusters were captured at 300 Hz by a Vicon MX optical motion capture system with eight Vicon F40 cameras (Vicon Motion System Ltd., LA, USA) (Figure 2A). In order to optimize resolution, accuracy and precision, the optical system used in this study included even more cameras than the previous validation study. The optical system was configured in line with our recent recommendations [46]. Specifications from our previous publication were followed, *i.e.*, positioning the cameras and adjusting the appropriate capture volume and the optimal distance between cameras and the tibia-affixed markers. It can therefore be taken as granted that the performance of the optical system was as good as in the mock-up study. This means that a resolution better than 20 µm within the capture volume of 400×300×300 mm^3^ was achieved. The maximal absolute error was 1.8 µm during displacements by 20 µm and repeatability was 2.5 µm. A detailed error analysis was performed in order to estimate absolute distance recording errors as a function of bending angle errors (see Discussion). Prior to the *in vivo* experiments, an *ex vivo* study on measuring tibia segment deformation under artificial loading in six cadaveric specimens has shown the fair repeatability and the feasibility of the OST approach (unpublished data). Briefly, the variance between the repeated tibia segment deformation measurements using the OST approach was assessed, whilst the cadaveric tibia was loaded by simulated muscle forces with a custom-made static loading device. Results suggested that the standard deviation of the mean bending and torsion deformation angles remains at a low level, from 0° to 0.04° for different loading conditions, indicating its potential to be applied *in vivo*. During the *in vivo* study, the stability of the bone screws in the tibial cortex was assessed by testing the resonance frequency of the screw-cluster structure and the relative position between the marker clusters prior to and after the exercises. It was shown to remain constant at ∼260 Hz and ∼380 Hz. The small location drift between the marker clusters during the course of the experimentation, which was maximally 0.06°, indicates that the implantation of the bone screws was extremely stable. The good toleration to the OST approach by the subjects also indicated its applicability for the *in vivo* measurements (See Results).

In order to identify the tibia anatomical landmarks, general retro-reflective markers (Ø16 mm, Vicon Motion System Ltd., LA, USA) were attached on the skin over the medial and lateral malleolus, the tibia tuberosity and over the head of the fibula. Prior to each trial of the activities, the trajectories of skin-attached markers were recorded simultaneously with tibia-affixed markers for 1–2 seconds while the shank was in a static position and free of any loading. This static trial allowed the generation of the Shank Anatomical Coordinate System (SACS) required for data analysis. Tibia segment deformation was expressed as the relative movement between the tibia-affixed marker clusters in the SACS.

### 1.3 Surgical technique

Surgical implantation and explantation of the bone screws was performed under local anesthesia by injecting Xylocain 1% and Carbostesin 0.5% into the skin and the periosteum of the right shank of each subject. Prior to the operation, Ibuprofen (600 mg) and Cefuroxime (1500 mg) was administered to reduce pain perception and the risk of infection. The thickness of the tibial cortex was determined from transverse MRI (Magnetic Resonance Imaging, 1.5 T, Philips, Best, The Netherlands) images of the shank. To prevent intrusion into the bone marrow, the sites for screw implantation were selected so that the thickness of the tibial cortex was thicker than 4 mm. Thus, screws were implanted at approximately 10 cm below the tibia plateau, in the middle of the tibia diaphysis and at approximately 10 cm above the tibia medial malleolus.

Surgical incisions of approximately 1 cm length were made into the skin. A drill guide and a 2.1 mm diameter drill (Stryker Leibinger GmbH & Co. KG, Germany) were used to drill three holes into the tibial cortex to a depth of 2.5 mm. Bone screws (Asnis Micro cannulated titanium screws, Ø3 mm, 24/6 mm, Stryker Leibinger GmbH & Co. KG, Germany) were implanted with a dedicated screw driver. At the end of the experiment, *i.e.* between 6 and 8 hours later, bone screws were removed. Further details of the surgical procedure were described in another paper [47].

### 1.4 Investigated locomotor activities

All of the subjects wore gymnastic shoes during the experiments. Members of staff familiarized with the experimental procedure once a week during 6 months preceding the study, and study subjects underwent preparatory training during at least one day prior to the actual experiments in order to be fully acquainted with the protocol. During the practice training, the experimental protocol was followed closely to help the test subjects to be mentally well prepared for the *in vivo* experiments – the exception being that bone screws were installed into a shin pad above the shank instead of into the tibia. Testing included the most common locomotor activities during daily life: (1) walking on a walkway with a force plate embedded at self-selected slow, free and fast speed, respectively (Figure 2A); (2) walking at 2.5, 3.5, 4.5 and 5.5 km/h on a treadmill (Schiller MTM-1500 med, h/p/cosmos sports & medical GmbH, Germany); (3) running at 5.5 and 9 km/h on a treadmill (Two test subjects participated in the running test at 9 km/h). At least three repetitions have been performed at each speed of walking on the walkway, and a minimum of sixteen complete walking or running cycles were recorded on the treadmill, respectively.

### 1.5 Assessment of speed and ground reaction forces during walking

A second, independent motion capturing system with ten Bonita cameras (Vicon Motion System Ltd., LA, USA) was installed for the assessment of whole-body movement. Two general retro-reflective markers (Ø16 mm, Vicon Motion System Ltd., LA, USA) were attached to the skin of the left and right posterior superior iliac spine (PSIS). The trajectories of these two markers were sampled at 100 Hz for all subjects. Ground reaction forces during walking on the walkway were recorded at 1000 Hz with a force plate system (AMTI OR6-5, Watertown, MA, USA). The two motion capturing systems were synchronized by an external trigger.

### 1.6 Peripheral quantitative computed tomography (pQCT) imaging

Horizontal pQCT scans of the tibia on the sites of screw implantation were obtained one to three days after screw removal with a XCT3000 (Stratec Medizintechnik, Pforzheim, Germany) to document the screw holes and geometry of the tibia cross section area for further calculations (Figure 2D).

### 1.7 Data analysis

Raw marker trajectory data and ground reaction forces were further processed with custom-written MATLAB routines (The MathWorks, Inc. Version 7.9.0 R2009b). The raw marker trajectory data for tibia segment deformation recording was filtered using a 10-point moving average filter. Ground reaction force data was low-pass filtered using a 2^nd^ order, zero lag Butterworth filter with cut-off frequency at 15 Hz.

#### 1.7.1 Determination of SACS, tibia bending and torsion angles

For each subject, a randomly selected frame acquired during the static trial was utilized to determine an initial Cartesian SACS from the skin-attached tibia landmarks. In the SACS, X, Y and Z axes indicate the anterior-posterior, proximal-distal and medial-lateral direction, respectively [48]. The coordinates of the marker clusters implanted into the tibia were then subjected to coordinate transformation [49], [50] to yield tibia segment deformation within the SACS. Differences between the relative position of each two sets of marker clusters in the SACS were then calculated and expressed as mean ± standard deviation (SD) of three Cardan/Euler angles and three translations along the axes of SACS, respectively. As the anterior-medial aspect of the tibia is free of muscle insertions, the effect of bone tissue inhomogeneity on anterior-medial tibia surface deformations was assumed to be negligible. AP bending angle, ML bending angle and inter-external torsion angle derived from Cardan/Euler angles were reported as tibia segment deformation. In the following, the relative movement of the proximal tibia-markers will be presented in relation to distal tibia-markers. Thus, AP bending (Figure 1B), ML bending and internal-external torsion (Figure 1C) always indicate the bending and torsion of the proximal tibia with respect to the distal tibia (Figure 1).

#### 1.7.2 Calculation of the ground reaction force

Vertical ground reaction force (VGRF) during walking was derived from the filtered ground reaction force data. In general, there are two noticeable peaks for the VGRF during walking. These two peak values of VGRF were automatically identified and used for further analysis. Vertical Free moment (VFM) is the torque which acts about the vertical axis through the center of pressure of the ground reaction force. By subtracting the AP moment and the ML moment from the total transverse ground reaction moment, VFM about the vertical axis through the center of pressure of the force plate was determined. The details on the moment calculation can be referred to the ‘Instruction Manual' of the AMTI Company [51]. VGRF was normalized to body weight (unit: N). VFM was normalized to the product of body weight (unit: N) and foot length (unit: m).

#### 1.7.3 Calculation of walking speed on the walkway

During walking trials on the walkway, the coordinates of the mid-point of two PSIS markers in global transverse plane were extracted from the filtered total trajectories data. The average walking speed of the subjects was determined as the moving distance of the mid-point of two PSIS markers divided by time over the stance phase of the right leg on the force plate.

### 1.8 Statistics

Statistical analyses were performed using R statistic software (version 2.15.1, R Development Core Team, 2012). Least-squares linear regression was performed to determine the correlation between the tibia segment deformation angles and the moving speed, as well as VGRF or VFM for individual subjects. The 95% confidence interval for the slope was calculated. Furthermore, a one-way ANOVA linear model was employed to examine the main effects of subject, moving speed and the type of activity on the tibia segment deformation angles. Within-subject effects due to moving speed were assessed with the error analyses in an ANOVA linear model. Furthermore, considering that the sample size in the present study was limited, nonparametric tests were also adopted to assess the potential influence of small sample size on the statistical results. In particular, the correlation between tibia segment deformation angles, moving speed and VGRF or VFM for each individual subject was determined with Spearman's rank correlation rho (corresponding to Least-squares Linear regression in parametric tests). The effects of locomotor speed and the type of activity on tibia segment deformation angles during treadmill exercises were assessed with Friedman test or Wilcoxon Mann-Whitney test (corresponding to one-way ANOVA and t-test). Statistical significance was accepted at *p*≤0.05.

## Results

Pain questionnaires (Visual analog scale form 0 to 10 indicating no pain to intolerable pain) were handed out during the *in vivo* experiments. All subjects report 0 during walking and running [47], indicating that the potential influence of pain caused by the bone screws and the wound on the motion is minimized. The bone screws were firmly inserted into the tibia until the end of the experiments. Statistical analysis suggested that there is no fundamental difference between parametric and nonparametric analysis. The details can be found in the following sections. The results in the corresponding figures were thus presented based on the parametric analysis.

### 1.1 Walking on the walkway with force plate embedded

**Figure 3:**
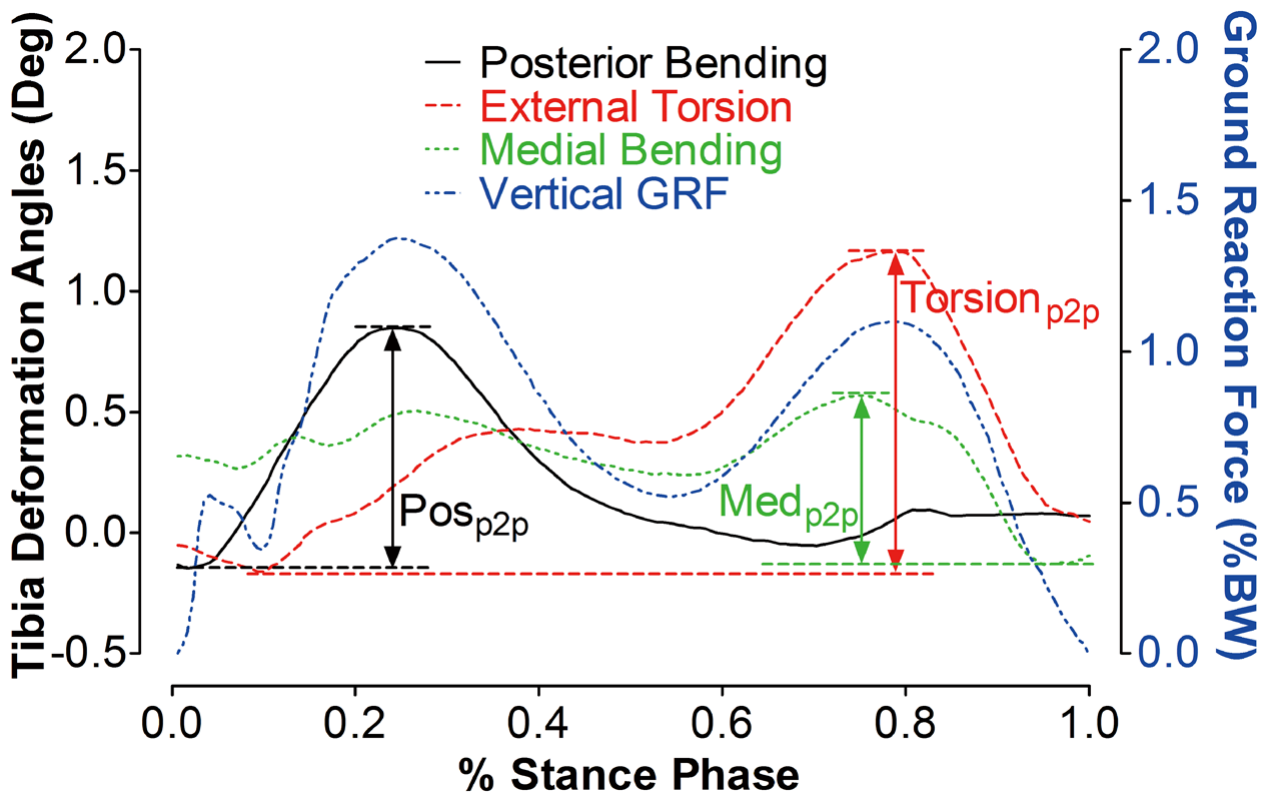
Illustration of the tibia segment deformation (example during the stance phase of an overground gait cycle). Solid black line: AP bending angle of the proximal tibia with respect to the distal tibia. Dashed red line: torsion angle, dotted green line: ML bending angle, dash-dot blue line: vertical ground reaction force. Pos_p2p_ refers to the peak-to-peak AP bending angle during the stance phase of the gait cycle. Med_p2p_ refers to the peak-to-peak ML bending angle of the proximal tibia. Torsion_p2p_ refers to the peak-to-peak torsion angle. AP: antero-posterior, ML: medio-lateral. Pos: posterior, Med: medial, p2p: peak-to-peak.

**Figure 4:**
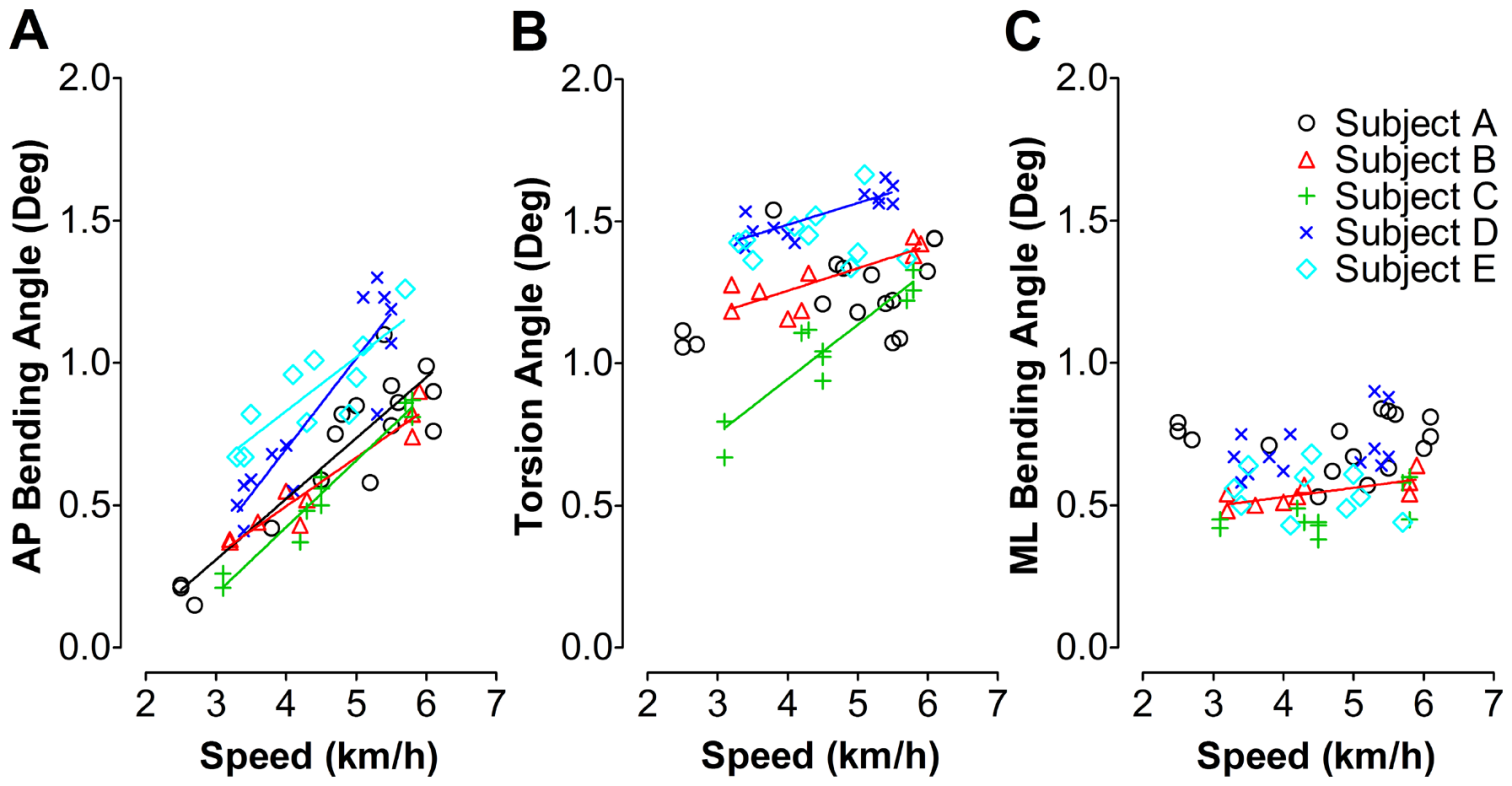
Illustration of individual tibia segment deformation during overground walking in relation to walking speed. The AP bending (A), torsion (B) and ML bending angle (C) indicate the extent of AP bending, external torsion and ML bending of the proximal tibia with respect to the distal tibia, respectively. The regression lines were given only when correlation between deformation angle and the walking speed was significant. It can be appreciated from these data that a high correlation exists between overground speed and bone deformation. This is despite the fact that locomotor patterns will contain elements that will not scale linearly with speed, which may underline the validity of the bone deformation measurements. AP: antero-posterior, ML: medio-lateral.

**Figure 5:**
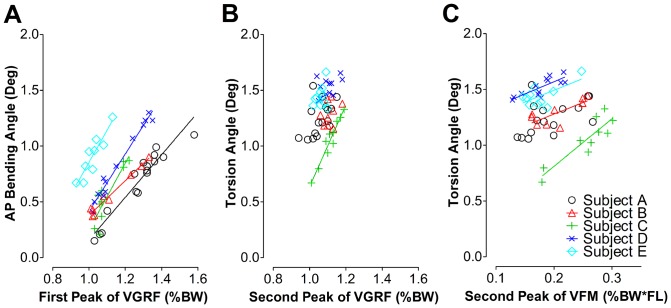
The relationship between the tibia segment deformation angles and the VGRF or VFM during walking. A: AP bending angles under different VGRF (the first peak value) during the first half stance phase of the gait cycle. B: Torsion angles under different VGRF (the second peak value) during the second half stance phase of the gait cycle. C: Torsion angles under different VFM during the second half stance phase of the gait cycle. The regression lines are displayed only where correlations between tibia deformation angles and the walking speed were significant. AP: antero-posterior, ML: medio-lateral. VGRF: vertical ground reaction force, VFM: vertical free moment.

**Figure 6:**
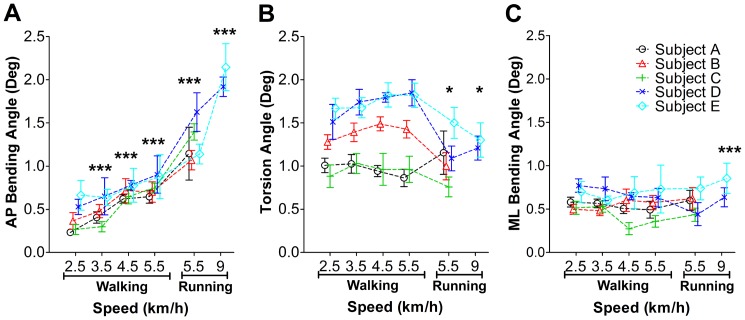
Tibia segment deformation angles during walking and running on a treadmill at different speed. A: tibia AP bending angles at different speed of walking and running. B: tibia torsion angle. C: tibia ML bending angle. *: *p*<0.05; ***: *p*<0.001. AP: antero-posterior, ML: medio-lateral.

A typical example of the tibia segment deformation angle during a stance phase of the gait cycle is presented in Figure 3. As the overground walking speed was not strictly controlled from subject to subject and computed after the experiments, the results in Figures 4–6 provide all data on an individual basis. During the stance phase, there were generally posterior bending, external torsion and medial bending of the proximal tibia relative to the distal tibia, with posterior bending being most pronounced during the first half, and torsion being predominant during the second half of the stance phase.

#### 1.1.1 Tibia segment deformation versus walking speed

Statistical analysis yielded main effects of the test subject on the deformation angles (*p* = 0.02 and r^2^ = 0.57 for tibia AP bending, *p*<0.001 and r^2^ = 0.09 for tibia torsion, *p*<0.001 and r^2^ = 0.03 for tibia ML bending). There were no significant within-subjects effects due to walking speed (*p* = 0.36 for tibia AP bending, *p* = 0.07 for tibia torsion, *p* = 0.1 for tibia ML bending). Therefore, the nonparametric and parametric statistic correlation analysis between deformation angles and walking speed was done separately for the individual test subjects (Figure 4). For all test subjects, the peak-to-peak AP bending angle linearly increased with walking speed. Peak-to-peak AP bending angles varied from 0.15° to 1.30° at the speed of 2.5 - 6.1 km/h. The slope of the regression line ranged from 0.17 to 0.32 with r-squared values between 0.70 and 0.96 (Figure 4A). Significant correlations between peak-to-peak tibia torsion angles and the speed were found in three out of five test subjects. The peak-to-peak tibia torsion angle varied between 0.67° and 1.66° at walking speed of 2.5–6.1 km/h (Figure 4B). By contrast, peak-to-peak ML bending angles were rather small, within 0.38°–0.90°, and were mostly un-related to walking speed, except for test subject B (Figure 4C). The linear regression results are summarized in Table 1. Nonparametric statistical analysis (Spearman's rank correlation) yielded the similar correlation results to the parametric analysis between tibia bending angles and walking speed. The results were summarized in Table 2.

**Table 1:** Least-squares linear regression statistics for tibia bending angles versus walking speed.

Subject	Deformation	b_1_ (degree*h/km)	a_1_ (degree)	r^2^	*p* value	N
A	AP Bending	0.21***	−0.33	0.82	<0.001	16
	Torsion	0.05	1.02	0.16	0.12	
	ML Bending	7.57e-4	0.72	9.90e-05	0.97	
B	AP Bending	0.17***	−0.19	0.92	<0.001	9
	Torsion	0.08**	0.94	0.68	0.006	
	ML Bending	0.03*	0.40	0.56	0.02	
C	AP Bending	0.23***	−0.52	0.96	<0.001	10
	Torsion	0.19***	0.18	0.85	<0.001	
	ML Bending	0.04	0.28	0.36	0.07	
D	AP Bending	0.32***	−0.58	0.82	<0.001	13
	Torsion	0.08***	1.19	0.71	<0.001	
	ML Bending	0.05	0.49	0.19	0.14	
E	AP Bending	0.19**	0.07	0.70	0.0027	10
	Torsion	0.01	1.39	0.01	0.80	
	ML Bending	−0.03	0.67	0.07	0.46	

The linear model used in the statistics is: y_1_ = b_1_ * x_1_+a_1_ (y_1_ indicates the tibia bending angles, x_1_ indicates walking speed, 95% Confident interval).

*: *p*<0.05,

**: *p*<0.01,

***: *p*<0.001.

**Table 2:** Nonparametric statistical analysis for tibia bending angles versus walking speed.

Subject	Deformation	r_s_	*p* value	N
A	AP Bending	0.77***	<0.001	16
	Torsion	−0.38	0.14	
	ML Bending	−0.22	0.42	
B	AP Bending	0.88**	0.003	9
	Torsion	−0.73*	0.03	
	ML Bending	−0.76*	0.02	
C	AP Bending	0.98***	<0.001	10
	Torsion	−0.77**	0.0098	
	ML Bending	−0.41	0.24	
D	AP Bending	0.78**	0.002	13
	Torsion	−0.74**	0.004	
	ML Bending	−0.40	0.17	
E	AP Bending	0.82**	0.004	10
	Torsion	−0.006	1	
	ML Bending	0.15	0.68	

The coefficient of correlation (r_s_) and level of significance (*p*) were yielded.

*: *p*<0.05,

**: *p*<0.01,

***: *p*<0.001.

#### 1.1.2 Tibia segment deformation versus ground reaction force

Regression analysis suggests that the tibia peak-to-peak AP bending angle increased linearly (*p*<0.001) with the peak VGRF during the first half stance phase, with the slope of the regression line ranging between 1.99 degree*h/km and 2.56 degree*h/km (r^2^: 0.77–0.97, Figure 5A). By contrast, there was no such relationship between peak-to-peak torsion angle and peak VGRF during the second half of the stance phase, except for subject C (*p*<0.001, r^2^ = 0.94, Figure 5B).

VFM but not VGRF was correlated with peak-to-peak torsion angles in four test subjects, except subject A (*p* = 0.056, r^2^ = 0.22, Figure 5C).

The linear regression results are summarized in Table 3. Similar correlation trends between the tibia bending angles and walking speed were found using nonparametric statistical analysis. The Spearman's rank correlation results are summarized in Table 4.

**Table 3:** Least-squares linear regression statistics for tibia deformation angles versus VGRF and VFM.

Subject	Deformation angles	b_3_ (degree*h/km)	a_3_ (degree)	r^2^	*p* value	N
A	AP Bending *v.s.* VGRF	1.91***	−1.75	0.91	<0.001	16
	Torsion *v.s.* VGRF	1.34	−0.17	0.24	0.054	
	Torsion *v.s.* VFM	11.97	1.07	0.25	0.056	
B	AP Bending *v.s.* VGRF	1.56***	−1.18	0.97	<0.001	9
	Torsion *v.s.* VGRF	1.02	0.16	0.11	0.38	
	Torsion *v.s.* VFM	28.43*	0.60	0.51	0.046	
C	AP Bending *v.s.* VGRF	2.99***	−2.74	0.77	<0.001	10
	Torsion *v.s.* VGRF	3.67***	−3.03	0.94	<0.001	
	Torsion *v.s.* VFM	34.91**	0.33	0.66	0.008	
D	AP Bending *v.s.* VGRF	2.57***	−2.16	0.97	<0.001	13
	Torsion *v.s.* VGRF	0.85	0.59	0.21	0.11	
	Torsion *v.s.* VFM	10.71**	1.31	0.42	0.003	
E	AP Bending *v.s.* VGRF	2.90***	−2.04	0.84	<0.001	10
	Torsion *v.s.* VGRF	1.05	0.33	0.08	0.43	
	Torsion *v.s.* VFM	16.52*	1.10	0.47	0.04	

The linear model used in the statistics is: y_3_ = b_3_ * x_3_+a_3_ (y_3_ indicates the tibia deformation angles, x_3_ indicates vertical ground reaction force or vertical free moment, 95% Confident interval).

*: *p*<0.05,

**: *p*<0.01,

***: *p*<0.001.

**Table 4:** Nonparametric statistical analysis for tibia bending angles versus VGRF and VFM.

Subject	Deformation angles	r_s_	*p* value	N
A	AP Bending *v.s.* VGRF	0.96***	<0.001	16
	Torsion *v.s.* VGRF	−0.53*	0.04	
	Torsion *v.s.* VFM	−0.52*	0.04	
B	AP Bending *v.s.* VGRF	0.85**	0.003	9
	Torsion *v.s.* VGRF	−0.23	0.55	
	Torsion *v.s.* VFM	−0.89*	0.012	
C	AP Bending *v.s.* VGRF	0.78**	0.007	10
	Torsion *v.s.* VGRF	0.92***	<0.001	
	Torsion *v.s.* VFM	−0.81**	0.008	
D	AP Bending *v.s.* VGRF	0.96***	<0.001	13
	Torsion *v.s.* VGRF	−0.41	0.17	
	Torsion *v.s.* VFM	−0.78**	0.002	
E	AP Bending *v.s.* VGRF	0.91***	<0.001	10
	Torsion *v.s.* VGRF	−0.16	0.66	
	Torsion *v.s.* VFM	−0.42	0.27	

The coefficient of correlation (r_s_) and level of significance (*p*) were yielded accordingly.

*: *p*<0.05,

**: *p*<0.01,

***: *p*<0.001.

### 1.2 Walking and running on a treadmill

During treadmill walking, significant main effects of the test subjects on the AP bending angle (*p*<0.001, r^2^ = 0.89), torsion angle (*p*<0.001, r^2^ = 0.85) and ML bending angle (*p* = 0.0046, r^2^ = 0.62) were found. Within-subjects effects of walking speed were not found (*p* = 0.24 for tibia AP bending, *p* = 0.37 for tibia torsion, *p* = 0.16 for tibia ML bending). Therefore, and as above, the deformation results are presented on the basis of the individual test subject (Figure 6). The AP bending angle increased with walking speed (posterior bending angles: from 0.23°±0.03° to 0.90°±0.22°, *p*<0.001) and running speed (posterior bending angles: from 1.07°±0.11° to 2.15°±0.27°, *p*<0.001). At the same speed, running induced a larger AP bending angle than walking (Figure 6A, *p*<0.001). No main effects of speed were found on tibia torsion during treadmill walking (external torsion angles: from 0.86°±0.10° to 1.85°±0.15 °, *p* = 0.067). Interestingly, for four test subjects, it seems that tibia torsion during running is lower than that during walking (Figure 6B, *p* = 0.048). During walking and running on the treadmill, ML bending, compared to bending deformation, occurred on somewhat low levels and was almost constant across speeds. One exception was that a larger ML bending angle was generated during running at 9 km/h than during running at 5.5 km/h and walking at different speeds (Figure 6C, *p*<0.001). Tibia torsion of two test subjects responded differently to the running speed. The tibia torsion angle significantly increased for test subject D (*p*<0.001), but decreased in test subject E (Figure 6B, *p* = 0.0013). The variation across the walking and running cycles was assessed by the standard deviation of the deformation angles, which was summarized in Table 5.

**Table 5:** The variation across the walking and running cycles was assessed with the standard deviation (SD) of the deformation angles.

			SD (Deformation Angles, Degree)		
Subject	Exercises	Cycles	AP Bending	Torsion	ML Bending
A	walking	5–19	0.03–0.07	0.08–0.10	0.06–0.12
	running	22	0.31	0.25	0.12
B	walking	22–15	0.10–0.20	0.08–0.13	0.06–0.13
	running	41	0.11	0.16	0.13
C	walking	23–48	0.06–0.08	0.13–0.19	0.06–0.08
	running	30	0.09	0.11	0.08
D	walking	6–39	0.09–0.22	0.05–0.20	0.08–0.14
	running	39–53	0.11–0.22	0.14	0.11–0.13
E	walking	16–39	0.09–0.25	0.12–0.17	0.05–0.25
	running	18–28	0.12–0.27	0.18–0.20	0.13–0.18

SD: standard deviation.

During treadmill walking, nonparametric analysis suggested a similar relationship between tibia deformation angles and moving speed as established during parametric analysis. More specifically, the AP bending angle increased with the walking speed (*p* = 0.007, except *p* = 0.42 for 2.5 km/h *v.s.* 3.5 km/h and *p* = 0.22 for 4.5 km/h *v.s.* 5.5 km/h comparisons). A larger AP bending angle was induced by running than walking (walking at 5.5 km/h *v.s.* run at 9 km/h: *p* = 0.03). No significant effects of speed were found on tibia torsion during treadmill walking (*p* = 0.22) and running (*p* = 0.08). ML bending remained at a low level and nearly constant across speeds (*p* = 0.45) and types of locomotion (*p* = 0.64).

## Discussion

In this paper, the *in vivo* tibia segment deformation regimes in humans, *e.g.* bending and torsion, during walking and running were investigated utilizing a novel optical segment tracking (OST) approach for the first time. Substantial effects of the walking and running speed, VGRF and VFM on the bending and torsion deformation angles of the human tibia were found. It should also be stated that these deformations were of surprisingly large magnitude during walking and running. In addition to the expected result that tibia segment deformation would generally increase with locomotor speed and with ground reaction forces, this study has yielded a number of novel and less obvious findings. Firstly, and most importantly, bone segment deformation, almost like a finger-print, contains highly specific personal information. In other words, very close relationships were found between *e.g.* ground reaction force and tibia segment deformation within each test subject, but the exact nature of these relationships varied between people. Secondly, anterior-posterior bending and torsion were the prevailing tibia loading regimes, whilst medio-lateral bending was much less pronounced. Thirdly, the different tibia deformation regimes did not scale uniformly with locomotor speed or ground reaction force. Each locomotor activity was rather characterized by a variable amount of bending and torsional deformation. Fourth, on the basis of many studies on the bone deformation amplitude in the past, this study provides rationale to revisit the potential importance of *in vivo* loading regimes and its features during common exercises, *e.g.* walking and running.

### 1.1 Analysis of recording errors

As with any new method, an assessment of limits arising from recording errors is vitally important. We see four major sources of error.

**Figure 7:**
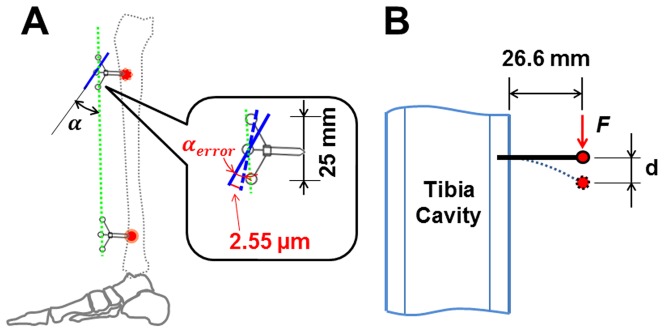
The recording error analysis of the OST approach. A: the deformation angle deviation α_error_ assumed from the absolute error of 2^1/2^ * 1.8 = 2.55 µm for both ends of the markers in the marker cluster. Bold black line referred to the plane determined by three markers in the marker cluster. 25 mm indicated the maximum distance between the markers in one marker cluster. α refers to the angle between the marker clusters. B: the potential marker displacement (d) due to the vibration induced by the acceleration force (*F*) of the screw/cluster structure. The bold black line refers to the bone screw. The red spot indicates the position of the plane determined by the markers in this cluster. 26.6 mm indicates the distance between the plane determined by three markers in one marker cluster and the bone surface. OST: optical segment tracking.

Firstly, the accuracy and the repeatability of the adopted optical system for recording minute marker movement in the targeted 3D volume have to be considered. As outlined above, the accuracy (absolute error) and the repeatability were very favorable within the volume of 400×300×300 mm^3^, namely maximum 1.8 µm and 2.5 µm, respectively, to assess displacements by 20 µm. The corresponding error can be translated in terms of angular deviation (α_error_ in Figure 7A) with the equations given as follows. The maximum between-marker distance within a marker cluster amounts to 25 mm, and we have to consider accuracy errors at both ends of the marker cluster. Thus, the total alignment error amounts to 2^1/2^ * 1.8 = 2.55 µm, and the error for estimates of α_error_ would be 180°–2 * arccos(2.55 µm/25 mm)  = 0.012° (the calculations of arccos were based on angles, Figure 7A). The α_error_ value was smaller than reported deformation results by two orders of magnitude.

Secondly, there is an undeterminable source of error associated with longitudinal variation as per the biological experiment itself. Our experience from the afore-mentioned *ex vivo* study suggested a reproducibility of approximately 0.04°. The value of 0.04° includes both measurement and experimental-longitudinal variation and is approximately twice as large as the measurement error only, but still substantially smaller than the reported results.

Thirdly, we have to consider that the bone screws could have loosened, *e.g.* due to the impact during the locomotor activities. However, the screws were deemed as stable upon removal after exercises were completed. Both orthopedic surgeons involved in the present study had performed hundreds of materials removals in their surgical practice. Moreover, the constant resonance frequency of the screw-cluster structure and the non-systematic and negligible drift between the marker clusters suggested firm fixation of the bone screws in the tibial cortex (unpublished data).

Fourthly, it is possible, in theory, that the marker cluster resonated during locomotor activities and thus produced artificial displacement. However, the vibration amplitude of the screw-cluster structure during the locomotor activities can be assessed with the known characteristics of the marker clusters and following equations, as illustrated in Figure 7B. The weight of the marker cluster is 5.6 grams. The most intense exercise, *i.e.* hopping, yielded acceleration of 3.5 times gravity, thus causing a force *F* of 0.19 N. The distance between the bone surface and the plane determined by three markers in the cluster is 26.6 mm (*L*), and the bending stiffness (flexural stiffness, *i.e.* the product of elastic modulus and area moment of inertia) of the bone screw shaft is 0.41 Nm^2^ (elastic modulus of the screw material: *E* = 110 GPa, inner and outer diameters of 1.5 and 3 mm, respectively, and thus area moment of inertia *I* = 3.73 mm^4^). Thus potential vibration amplitude of the markers induced by the acceleration of the marker cluster would be maximally [52]

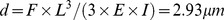



These results suggested that the amplitude of any vibration of the screw/cluster structure is relatively small, being certainly smaller than the resolution of the optical system, and probably negligible in comparison to the reported results.

### 1.2 Main findings on tibia segment deformation during walking on the walkway

Results from this study showed that the proximal tibia mainly twisted externally and bent to the posterior aspect, as well as to some extent to the medial aspect in relation to the distal tibia during the stance phase of walking and running. Previously, the tibia contact force and moment have been investigated with an instrumented knee implant [31], [45]. These results are in accordance with our findings, especially regarding the occurrence of posterior and medial bending as well as torsion moments during the stance phase of level walking. In line with this, bending and torsion moments have been predicted to occur during walking in a musculoskeletal model calculation [30]. However, the results in our study disagree with previous reports of tibia bending assessed during running by inverse dynamics analysis, in which anterior, rather than posterior tibia bending moments were postulated during the stance phase of running [53]. The inconsistencies may relate to the inherent limitations of the inverse dynamics analysis approach, *e.g.* only joint reaction forces are calculated, which may derive unrealistic tibia load. In this context, the present results may provide further, indirect evidence to the view that the largest skeletal forces depend on muscle contractions, rather than simply arising from mass acceleration [54].

Another important finding is the non-uniform scaling of deformation regimes. As exemplified in Figure 3, there were generally two noticeable deformation peaks, one in antero-posterior bending that coincided with the heel-strike, and another one in torsion that coincided with toe lift-off. This pattern was also confirmed by statistical analyses, suggesting that the peak-to-peak antero-posterior bending angles are linearly correlated with the first peak of the vertical ground reaction force, while the peak-to-peak torsion angle is unrelated to the second peak of the VGRF, but correlates with the second peak of VFM during the second half of the stance phase. Considering the fact that the mechanical load on the tibia shaft is generally caused by body weight and muscle contractions, the plantar flexors are primarily active during the second half of the stance phase. It is therefore tempting to assume that the body weight primarily induces the posterior bending of the tibia, while torsion is mainly produced by the plantar flexors contraction. Certainly, further study into the relationship between the deformation angles and body weight or muscle activities is needed to draw a firm conclusion.

### 1.3 Effects of walking speed on tibia segment deformation

During walking on the walkway, tibia antero-posterior bending increased linearly with walking speed. This result is congruent with previous results of numerous animal experiments, *e.g.* dog tibia, dog radius, horse tibia, horse radius and goat tibia [21], [55]. Similarly, for three test subjects, the torsion angles, but not the medio-lateral bending angles, slightly and linearly increased with walking speed. The medio-lateral bending angles remained rather constant with speed. Likewise, during treadmill walking, antero-posterior bending increased with speed. Interestingly, for three test subjects, tibia torsion angles increased linearly with walking speed during overground walking, but remained constant during treadmill walking, indicating that the tibia load might be different for these two cases. It has been shown that larger compression and tension strains on one site of bone were generated during the overground running than during the treadmill running [56]. The current results provide further evidence that the VGRF during the mid- and late-stance phase of treadmill walking differs from overground walking [57], which might be able to explain the deformation difference found in the present study.

### 1.4 Relationship between tibia segment deformation and ground reaction force or moment

The results from this study revealed a strong relationship between the VFM and the tibia torsion deformation for four of the test subjects. It is of interest in this context that VFM seems to be closely related to the loading history of the tibia [58]. Conversely, the results showed that tibia deformation or load could not be totally predicted from VGRF.

### 1.5 Tibia segment deformation during running on the treadmill

In general, running is a more demanding exercise than walking. The muscles in the lower extremities are generally more active during running than during walking. In addition, axial forces caused by mass acceleration are higher in running than in walking. Results from this study suggest that the antero-posterior bending angle during running is significantly larger than during walking, even at the same speed of locomotion (Figure 6). Despite the limited number of subjects (n = 2) participating in the jogging trials at 9 km/h, antero-posterior and medio-lateral bending were still significantly larger than at 5.5 km/h. Conversely, tibia torsion was profoundly decreased during jogging at 9 km/h, even below levels observed during walking. Previous strain gauge measurements generally found principal tibia strains to be larger during running than walking. However, no measurements in bending and torsion were available in these previous studies [59], [60].

Taken all of the above together, results of the present study indicated that not only the amplitude, but the regimes of tibia load differ between running and walking. It has been well accepted that stress fractures in the anterior tibia shaft can occur among long distance runners [61]. Our experiment suggests that such stress fractures may be related to the high tension in the anterior aspect of tibia, whilst the posterior tibia was under even larger compression. Likewise, an inverse dynamics analysis study on runners indicated that the superposition of the joint reaction force and muscle force magnify the tibia posterior compression and attenuated the tibia shear force [62]. This might be one of the reasons why torsion angles from our measurements were lower during running than walking.

### 1.6 Limitations

Although new knowledge on human tibia segment deformation was contributed to understand the *in vivo* loading situations, the OST approach leaves some open questions. Firstly, unlike the strain gauge approaches, the local strain information of the tibia surface is not assessed in the proposed approach. Such a high-fidelity estimation or calculation should rely on an inversely-driven Finite Element Model (FEM), with anatomical tibia data – an approach that is by no means trivial but necessary to understand the strain distribution across the tibia. Secondly, the capture volume of the optical system was limited (400×300×300 mm^3^ in this case) in order to maintain acceptable accuracy and repeatability during the deformation recording, meaning that tibia-affixed markers have to be in this volume during the recording trials of the exercises. As a consequence, the selection of exercises which can be performed, *e.g.* continuous recording of long term walking (Even for single gait cycle, the full swing phase is not always available due to the restriction of the capture volume) or running over ground, is limited. Thirdly, in the presented study, the comparatively small sample size (n = 5) raises the issue of interpretation of the parametric statistical analysis. Hence, nonparameteric statistics, which are conceived to be more robust for small sample size, were used in addition to parametric statistics to analyse the present results. No clear differences were found when comparing the two types of analyses, indicating that the small sample size in the present study is not likely to influence the conclusions we drew form the results. However, a larger sample size would still be appreciated in future studies. To summarize, it remains uncertain in how far the OST approach will be applied widely in future studies, due to its invasiveness. Understanding what can and what cannot be expected from the OST approach will guide the design of future studies, which firstly need to focus on improvement of the OST approach, and then further expand its application when justifiable.

## Conclusions

In summary, taking together the tibia segment deformation results from this first application of the proposed OST approach in humans *in vivo*, we conclude that the human tibia experiences a considerable amount of bending and torsion loading during walking and running. The maximum peak-to-peak antero-posterior bending, torsion and medio-lateral bending angles reached up to 1.30°, 1.66° and 0.90° during walking, respectively. The tibia antero-posterior bending angles and torsion angles increased linearly with the walking speed and VGRF or VFM. More interestingly, a more or less fixed phase-relationship exists between different types of deformation during the stance phase of walking. Running generates larger antero-posterior bending angles, but smaller torsion angles than walking. These new findings on tibia segment deformation regimes during walking and running are therefore bound to change our understanding of long bone deformation in humans and provide more insights into the mechanical load distribution rather than mechanical load amplitude alone.
